# Comprehensive Search for GPCR Compounds which Can Enhance MafA and/or PDX-1 Expression Levels Using a Small Molecule Compound Library

**DOI:** 10.1155/2023/8803172

**Published:** 2023-09-08

**Authors:** Hideaki Kaneto, Atsushi Obata, Masashi Shimoda, Tomohiko Kimura, Yoshiyuki Obata, Tomoko Ikeda, Saeko Moriuchi, Shuhei Nakanishi, Tomoatsu Mune, Kohei Kaku

**Affiliations:** ^1^Department of Diabetes, Endocrinology and Metabolism, Kawasaki Medical School, Japan; ^2^Kawasaki Medical School General Medical Center, Japan

## Abstract

It has been shown that chronic hyperglycemia gradually decreases insulin biosynthesis and secretion which is accompanied by reduced expression of very important insulin gene transcription factors MafA and PDX-1. Such phenomena are well known as *β*-cell glucose toxicity. It has been shown that the downregulation of MafA and/or PDX-1 expression considerably explains the molecular mechanism for glucose toxicity. However, it remained unknown which molecules can enhance MafA and/or PDX-1 expression levels. In this study, we comprehensively searched for G protein-coupled receptor (GPCR) compounds which can enhance MafA and/or PDX-1 expression levels using a small molecule compound library in pancreatic *β*-cell line MIN6 cells and islets isolated from nondiabetic C57BL/6 J mice and obese type 2 diabetic C57BL/KsJ-db/db mice. We found that fulvestrant and dexmedetomidine hydrochloride increased MafA, PDX-1, or insulin expression levels in MIN6 cells. We confirmed that fulvestrant and dexmedetomidine hydrochloride increased MafA, PDX-1, or insulin expression levels in islets from nondiabetic mice as well. Furthermore, these reagents more clearly enhanced MafA, PDX-1, or insulin expression levels in islets from obese type 2 diabetic db/db mice in which MafA and PDX-1 expression levels are reduced due to glucose toxicity. In conclusion, fulvestrant and dexmedetomidine hydrochloride increased MafA, PDX-1, or insulin expression levels in MIN6 cells and islets from nondiabetic mice and obese type 2 diabetic db/db mice. To the best of our knowledge, this is the first report showing some molecule which can enhance MafA and/or PDX-1 expression levels. Therefore, although further extensive study is necessary, we think that the information in this study could be, at least in part, useful at some point such as in the development of new antidiabetes medicine based on the molecular mechanism of *β*-cell glucose toxicity in the future.

## 1. Introduction

Under diabetic conditions, when pancreatic *β*-cells are chronically exposed to hyperglycemia, *β*-cells are compelled to continuously secrete larger amounts of insulin to reduce blood glucose levels. Consequently, *β*-cell function is gradually deteriorated under diabetic conditions. Such phenomena are well known as *β*-cell glucose toxicity [1–8]. It has been shown that chronic hyperglycemia gradually decreases insulin biosynthesis and secretion which is accompanied by reduced expression of very important insulin gene transcription factors MafA and PDX-1 [9–13]. MafA strongly transactivates the insulin gene through a *cis*-regulatory element named RIPE3b1, and thereby MafA is particularly important for the preservation of mature *β*-cell function such as insulin biosynthesis and secretion [14–18]. PDX-1 also plays a crucial role in the maintenance of mature *β*-cell function as well as in pancreas development and *β*-cell differentiation [19–23]. It has been also shown that *β*-cell function is recovered by the reduction of glucose toxicity with various antidiabetes medicines which is also accompanied by the recovery of MafA and PDX-1 expression [24–27]. In addition, *β*-cell-specific transgenic overexpression of MafA or PDX-1 in diabetic mice preserved insulin biosynthesis and secretion, thus ameliorating glycemic control [28, 29]. These data clearly indicate that the downregulation of MafA and/or PDX-1 expression considerably explains the molecular mechanism for *β*-cell glucose toxicity. It remained unknown, however, which molecules can enhance MafA and/or PDX-1 expression levels without the reduction of glucose toxicity or the usage of transgenic techniques. In addition, it has been drawing much attention recently that G protein-coupled receptor (GPCR) signalling plays crucial roles in various kinds of cells including pancreatic *β*-cells [30–37]. The aim of this study was to comprehensively search for GPCR compounds which can enhance MafA and/or PDX-1 expression levels.

## 2. Methods

### 2.1. Materials

A small molecule compound library including 278 GPCR compounds (catalogue number: L2200) was purchased from Selleck so that we can comprehensively search for GPCR compounds which can enhance MafA and/or PDX-1 expression levels. Each GPCR compound was dissolved in a reagent which was thought to be appropriate for each compound.

### 2.2. Cell Culture

Pancreatic *β*-cell line MIN6 cells were cultured in modified DMEM (glucose concentration: 1,000 mg/L) at 37°C under humidified 5% CO_2_/95% air conditions.

### 2.3. Animals

We purchased nondiabetic C57BL/6 J and type 2 diabetic C57BL/KsJ-db/db mice from CLEA (Tokyo). This study was approved by the Animal Use Committee of Kawasaki Medical School (No. 18-023). This study was conducted in accordance with the Animal Use Guidelines of the Kawasaki Medical School.

### 2.4. Isolation of Pancreatic Islets from Mice

Isolation of pancreatic islets from mice was conducted as previously reported [27, 38]. After ligation of the common bile duct, Hanks' balanced salt solution (Sigma) containing 0.6 mg of Liberase TL (Roche) was injected into the duct. We removed the swollen pancreas and incubated it at 37°C for 24 min. After dispersing the pancreatic tissue by pipetting, we manually picked up islets under a stereoscopic microscope.

### 2.5. Treatment with GPCR Compounds in Isolated Islets

After isolation of pancreatic islets from 12-week-old nondiabetic C57BL/6 J mice, we treated the isolated islets with 0-50 *μ*M of each GPCR compound for 24 hours and examined the effects of each compound on *β*-cell-specific gene expression such as MafA, PDX-1, insulin 1, and insulin 2. Similarly, after isolation of islets from 12-week-old obese type 2 diabetic C57BL/KsJ-db/db mice, we treated isolated islets with 0-50 *μ*M of each GPCR compound for 24 hours and examined the effects of each compound on the abovementioned gene expression.

### 2.6. RNA Preparation and Quantitative PCR

Total RNA was extracted from isolated islets using the RNeasy Mini Kit (Qiagen), and cDNA was produced using TaqMan reverse transcription reagents (Applied Biosystems). We performed quantitative PCR using a 7500 real-time PCR system, as previously reported [27, 38]. The primer sequences of MafA, PDX-1, insulin 1, and insulin 2 are as follows:

MafA: (forward) CCAGCTGGTATCCATGTCC.

(reverse) TTCTGTTTCAGTCGGATGACC.

PDX-1: (forward) CGGCTGAGCAAGCTAAGGTT.

(reverse) TGGAAGAAGCGCTCTCTTTGA.

Insulin 1: (forward) CCCTTAGTGACCAGCTATAATCAGAGA.

(reverse) ACCACAAAGATGCTGTTTGACAA.

Insulin 2: (forward) CTGCTGGCCCTGCTCTTC.

(reverse) AACCACAAAGGTGCTGCTTGA.

*β*-Actin: (forward) CTAAGGCCAACCGTGAAAAG.

(reverse) ACCAGAGGCATACAGGGACA.

### 2.7. Statistics

Results are shown as mean ± SDs. We examined differences between the two groups using the Student's *t*-test. The effects of the compounds were evaluated using the multiple comparison method (ANOVA). We considered *p* < 0.05 statistically significant.

## 3. Results

### 3.1. Comprehensive Search for GPCR Compounds which Can Enhance MafA and/or PDX-1 Expression Levels in Pancreatic *β*-Cell Line MIN6 Cells

**Figure 1:**
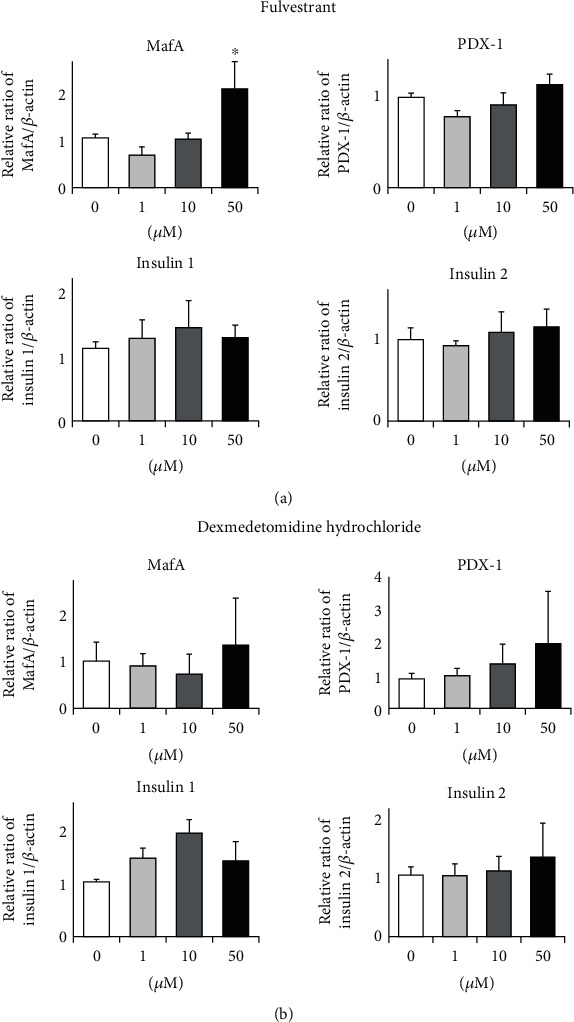
(a) Effects of fulvestrant on MafA, PDX-1, insulin 1, and insulin 2 mRNA expression levels in pancreatic *β*-cell line MIN6 cells. (b) Effects of dexmedetomidine hydrochloride on MafA, PDX-1, insulin 1, and insulin 2 mRNA expression levels in *β*-cell line MIN6 cells (*n* = 6). ^∗^*p* < 0.05.

**Figure 2:**
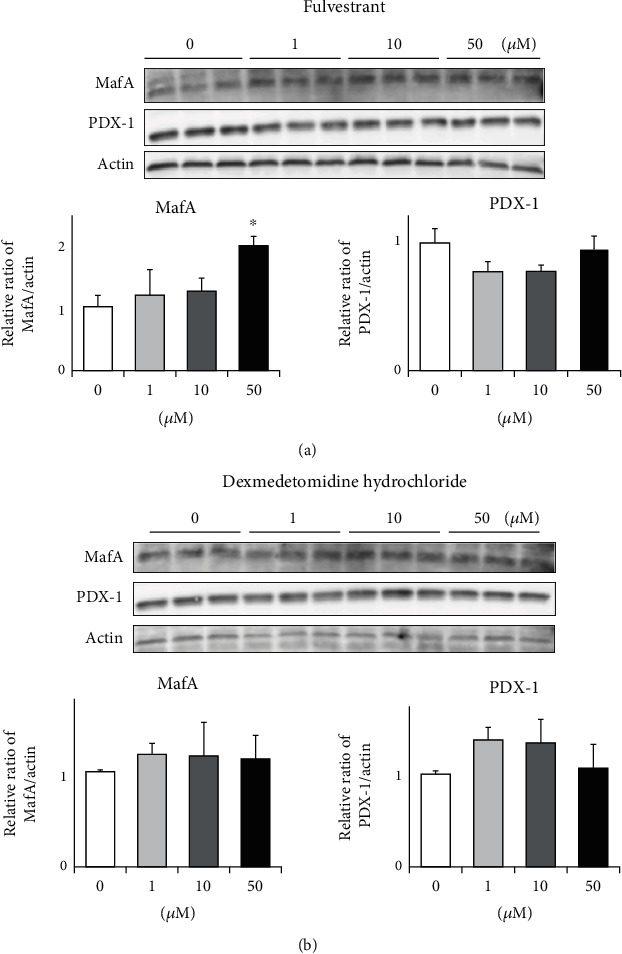
(a) Effects of fulvestrant on MafA, PDX-1, insulin 1, and insulin 2 protein expression levels in pancreatic *β*-cell line MIN6 cells. (b) Effects of dexmedetomidine hydrochloride on MafA, PDX-1, insulin 1, and insulin 2 protein expression levels in *β*-cell line MIN6 cells (*n* = 6). ^∗^*p* < 0.05.

To search for GPCR compounds which can enhance MafA and/or PDX-1 expression levels, we performed comprehensive analyses using a small molecule compound library including 278 GPCR compounds in pancreatic *β*-cell line MIN6 cells. First, after MIN6 cells were treated for 24 hours with 0 *μ*M, 1 *μ*M, 10 *μ*M, and 50 *μ*M of each GPCR compound, we examined MafA, PDX-1, insulin 1, and insulin 2 expression using RT-PCR. We performed this step for a total of 278 small molecules sequentially. Second, we searched for molecules which increased MafA or PDX-1 expression levels by over twofold at 50 *μ*M in the average of 3 experiments. Finally, we picked up two molecules: fulvestrant and dexmedetomidine hydrochloride. As shown in Figure 1(a), 50 *μ*M of fulvestrant increased MafA expression level by over twofold, which was significantly higher compared to that without treatment. In addition, as shown in Figure 1(b), 50 *μ*M of dexmedetomidine hydrochloride increased PDX-1 expression level by over twofold, although it did not reach a statistically significant difference. Fulvestrant is a ligand of the estrogen receptor which selectively degrades the estrogen receptor. This medicine has been used to treat estrogen receptor-positive breast cancer in clinical practice. Dexmedetomidine hydrochloride is a ligand of the adrenaline receptor which inhibits the release of norepinephrine from synaptic vesicles. This medicine has also been used for analgesia, sedation, and antianxiety in clinical practice. Next, we examined the effects of fulvestrant and dexmedetomidine hydrochloride on MafA and PDX-1 protein expression levels in MIN6 cells. As the results, 50 *μ*M of fulvestrant significantly increased MafA protein level (Figure 2(a)), although there was no statistically significant difference in PDX-1 protein expression level between with and without dexmedetomidine hydrochloride treatment (Figure 2(b)).

### 3.2. Effects of Two GPCR Compounds Fulvestrant and Dexmedetomidine Hydrochloride on MafA and/or PDX-1 Expression in Pancreatic Islets Isolated from Nondiabetic Mice

**Figure 3:**
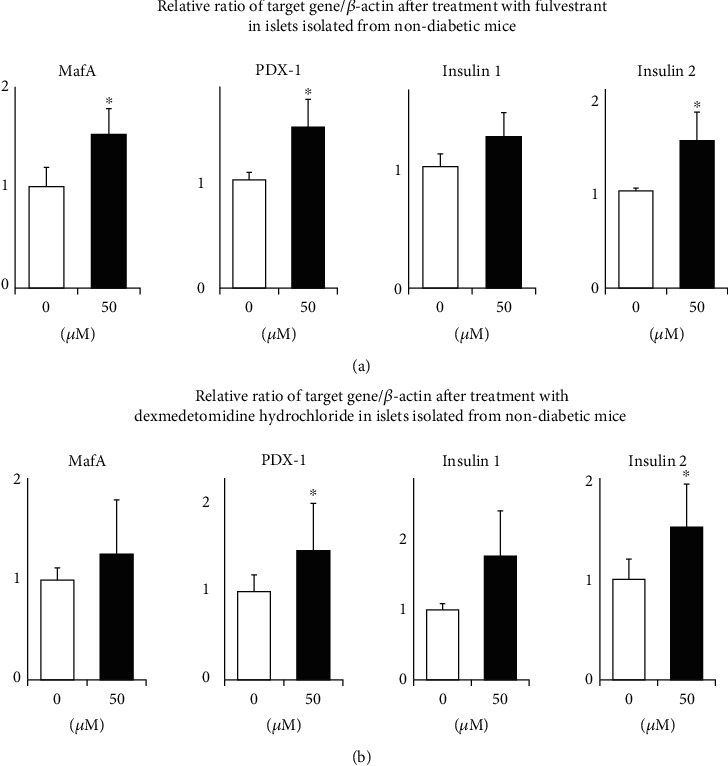
(a) Effects of fulvestrant on MafA, PDX-1, insulin 1, and insulin 2 expression levels in pancreatic islets isolated form nondiabetic C57BL/6 J mice. (b) Effects of dexmedetomidine hydrochloride on MafA, PDX-1, insulin 1, and insulin 2 expression levels in islets isolated from nondiabetic mice (*n* = 6). ^∗^*p* < 0.05.

As described above, we used MIN6 cells as a screening to comprehensively search for GPCR compounds which can enhance MafA and/or PDX-1 expression levels. It is known well, however, that since MIN6 cells are an immortalized pancreatic *β*-cell line, the characteristics of MIN6 cells are different from those of isolated pancreatic islets. Therefore, to confirm that the data obtained in MIN6 cells are true in isolated pancreatic islets, we performed similar experiments with the abovementioned two compounds fulvestrant and dexmedetomidine hydrochloride in pancreatic islets isolated from 12-week-old nondiabetic C57BL/6 J mice. As shown in Figure 3(a), in islets isolated from nondiabetic mice, 24-hour treatment with 50 *μ*M of fulvestrant significantly increased both MafA and PDX-1 expression levels in islets isolated from nondiabetic mice (1.53 ± 0.25-fold, *p* < 0.05 and 1.49 ± 0.26-fold, *p* < 0.05, respectively) (mean ± SD in 3 experiments). In addition, fulvestrant significantly increased insulin 2 expression level (1.52 ± 0.29-fold, *p* < 0.05). Also, as shown in Figure 3(b), in islets isolated from nondiabetic mice, 24-hour treatment with 50 *μ*M of dexmedetomidine hydrochloride significantly increased PDX-1 expression level (1.46 ± 0.54-fold, *p* < 0.05). In addition, dexmedetomidine hydrochloride significantly increased insulin 2 expression level (1.52 ± 0.43-fold, *p* < 0.05).

### 3.3. Effects of Two GPCR Compounds Fulvestrant and Dexmedetomidine Hydrochloride on MafA and/or PDX-1 Expression in Pancreatic Islets Isolated in Type 2 Diabetic Mice where MafA and PDX-1 Expression Levels Are Substantially Reduced due to Glucose Toxicity

**Figure 4:**
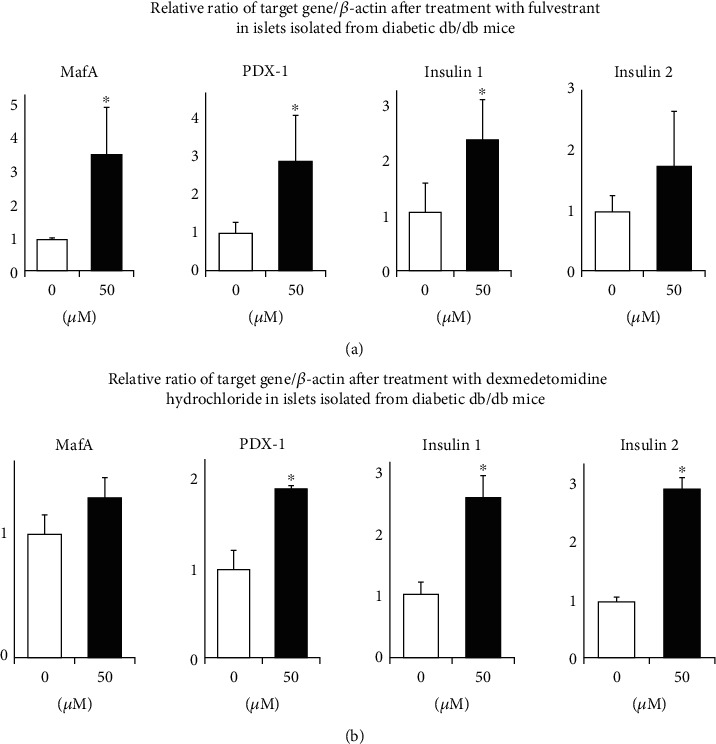
(a) Effects of fulvestrant on MafA, PDX-1, insulin 1, and insulin 2 expression levels in pancreatic islets isolated form obese type 2 diabetic C57BL/KsJ-db/db mice. (b) Effects of dexmedetomidine hydrochloride on MafA, PDX-1, insulin 1, and insulin 2 expression levels in islets isolated from type 2 diabetic mice (*n* = 6). ^∗^*p* < 0.05.

MafA and PDX-1 are abundantly expressed under healthy conditions, but their expression levels are substantially reduced under diabetic conditions due to glucose toxicity. Therefore, we thought that these compounds would show more clear effects on MafA and PDX-1 expression in islets isolated from diabetic mice compared to nondiabetic mice, and we performed the same experiments with the abovementioned two compounds fulvestrant and dexmedetomidine hydrochloride in islets isolated from 12-week-old obese type 2 diabetic C57BL/KsJ-db/db mice. As shown in Figure 4(a), in islets isolated from diabetic db/db mice, 24-hour treatment with 50 *μ*M of fulvestrant significantly increased MafA and PDX-1 expression levels (3.75 ± 1.53-fold, *p* < 0.05 and 2.94 ± 1.24-fold, *p* < 0.05, respectively). In addition, fulvestrant significantly increased insulin 1 expression level (2.24 ± 0.69-fold, *p* < 0.05). Also, as shown in Figure 4(b), in islets isolated from diabetic db/db mice, 24-hour treatment with 50 *μ*M of dexmedetomidine hydrochloride significantly increased PDX-1 expression level (1.92 ± 0.03-fold, *p* < 0.05). In addition, dexmedetomidine hydrochloride significantly increased insulin 1 and insulin 2 expression levels (2.54 ± 0.50-fold, *p* < 0.05 and 1.76 ± 0.21-fold, *p* < 0.05, respectively). As expected, the degree of increase in MafA, PDX-1, or insulin expression by fulvestrant or dexmedetomidine hydrochloride was larger in islets isolated from diabetic mice compared to those from nondiabetic mice.

## 4. Discussion

Chronic hyperglycemia gradually decreases insulin biosynthesis and secretion which is accompanied by reduced expression of very important insulin gene transcription factors MafA and PDX-1 [9–13]. It has been reported that the downregulation of MafA and/or PDX-1 expression considerably explains the molecular mechanism for *β*-cell glucose toxicity [28, 29]. It remained unknown, however, which molecules can enhance MafA and/or PDX-1 expression levels. In addition, it has been drawing much attention recently that G protein-coupled receptor (GPCR) signalling plays crucial roles in various kinds of cells including pancreatic *β*-cells [30–37].

In this study, we showed that fulvestrant and dexmedetomidine hydrochloride increased MafA and PDX-1 expression in MIN6 cells (Figures 1 and 2) and pancreatic islets isolated from nondiabetic and type 2 diabetic mice (Figures 3 and 4). To the best of our knowledge, this is the first report showing some molecule which can directly enhance MafA and/or PDX-1 expression levels. Insulin 1 or 2 expression level was also increased by fulvestrant or dexmedetomidine hydrochloride (Figures 3 and 4). These data indicate the possibility that increased MafA and/or PDX-1 expression functioned in isolated islets, although we cannot exclude the possibility that these compounds increased insulin expression levels. Additionally, as we expected, the increase in MafA, PDX-1, or insulin expression by fulvestrant or dexmedetomidine hydrochloride was more clearly observed in islets isolated from diabetic mice (Figure 4). MafA and/or PDX-1 expression levels are substantially reduced due to glucose toxicity which we assume can explain the reason why the two reagents more clearly enhanced MafA and/or PDX-1 expression levels in islets isolated from diabetic mice.

There are several limitations to this study. First, although the effects of fulvestrant or dexmedetomidine hydrochloride on MafA and/or PDX-1 mRNA levels in islets isolated from type 2 diabetic db/db mice were more clearly observed, we failed to show the possible effects of these compounds on MafA and/or PDX-1 protein levels due to the difficulty of obtaining enough amounts of protein from diabetic db/db mice with which we can perform western blotting. Second, since fulvestrant and dexmedetomidine hydrochloride substantially increased MafA and/or PDX-1 expression levels together with increased expression of insulin in islets isolated from type 2 diabetic mice, we assumed that these compounds would have possible glucose-lowering effects in type 2 diabetic mice. Based on such speculation, we performed direct injection of each compound into type 2 diabetic mice, but glucose-lowering effects were not clearly observed. We assume that there are several hurdles which we will have to get over such as the improvement of absorption efficiency of each compound in the body. Also, we assume that some chemical modification of these compounds would be necessary for each compound to obtain glucose-lowering effects in type 2 diabetic mice and to be utilized as an antidiabetic drug in clinical practice.

To the best of our knowledge, this is the first report showing some molecule which can enhance MafA and/or PDX-1 expression levels. Since both compounds have already been used in clinical practice for some purpose, it seems that their safety is guaranteed to some extent. Therefore, although further extensive study is necessary to strengthen our hypothesis, we think that the information in this study could be, at least in part, useful at some point such as in the development of new antidiabetes medicine based on the molecular mechanism for *β*-cell glucose toxicity in the future.

## Data Availability

The datasets generated and/or analyzed during the current study are available from the corresponding author on reasonable request.
